# Insight into the Prospects of RNA Interference for Honey Bee Pathogens and Parasite Control

**DOI:** 10.3390/insects17060646

**Published:** 2026-06-18

**Authors:** A-Tai Truong, Mi-Sun Yoo, Khanh Linh Ha Tran, So Youn Youn, Hyang-Sim Lee, Yun Sang Cho

**Affiliations:** 1 Department of Biotechnology, Faculty of Natural Sciences and Technology, Thai Nguyen University of Sciences, Thai Nguyen 250000, Vietnam; taita@tnus.edu.vn (A.-T.T.); halinhlc1711@gmail.com (K.L.H.T.); 2 Bacterial Disease Division, Department of Animal and Plant Health Research, Animal and Plant Quarantine Agency, Gimcheon-si 39660, Gyeongsangbuk-do, Republic of Korea; msyoo99@korea.kr (M.-S.Y.); syyoun@korea.kr (S.Y.Y.)

**Keywords:** RNA interference, *Varroa destructor*, small hive beetle, *Nosema ceranae*, honey bee viral disease

## Abstract

Honey bee populations face significant threats from viral pathogens, *Nosema ceranae*, *Varroa destructor*, and the small hive beetle (*Aethina tumida*), all of which contribute to colony losses worldwide. RNA interference (RNAi) has emerged as a promising molecular tool for controlling these pathogens and pests through sequence-specific gene silencing. This review summarizes current advances in RNAi applications against major honey bee diseases and parasites, including antiviral strategies, suppression of *Nosema* development, interference with *Varroa* reproduction, and RNAi-based control of small hive beetles. Particular attention is given to recent developments in delivery technologies, including oral administration, nanoparticle-assisted formulations, and symbiont-mediated RNAi systems. The opportunities, limitations, and future challenges associated with large-scale implementation, environmental safety, regulatory approval, and field deployment are also discussed. Collectively, these advances highlight the potential of RNAi as a valuable component of sustainable honey bee health management and integrated pest management programs.

## Simple Summary

Honey bee populations are increasingly threatened by viruses, microsporidian parasites, mites, and invasive pests, resulting in substantial ecological and economic impacts. RNA interference (RNAi) has emerged as a highly specific method for suppressing these threats by targeting essential genes in pathogens and parasites. This review summarizes recent progress in RNAi-based approaches for honey bee health management and discusses emerging delivery technologies that may facilitate future field applications.

## 1. Introduction

Honey bees are essential to the health and functioning of terrestrial ecosystems, serving as the primary pollinators of flowering plants [1]. Through pollinating a broad spectrum of wild and cultivated plants, honey bees maintain ecological balance and support biodiversity. Their pollination services are indispensable to agricultural systems, facilitating the production of approximately one-third of the food crops consumed globally, including fruits, vegetables, nuts, and oilseeds [2]. In the United States alone, the economic value of honey bee pollination is estimated at $10~20 billion annually, with some studies suggesting contributions up to $24 billion when indirect benefits to industries such as food processing, transport, and export are considered [2]. Beyond their ecological and agricultural significance, honey bees also produce a variety of economically valuable products, including honey, propolis, bee venom, royal jelly, and beeswax, which have applications in the food, pharmaceutical, and cosmetic industries.

Despite their critical importance, honey bee populations have experienced substantial declines over recent decades [3,4,5]. In the United States, annual colony losses have frequently exceeded 30%, and recent nationwide surveys reported losses surpassing 50–60% in some commercial beekeeping operations, highlighting the escalating threat to apiculture and pollination services [6]. Colony declines are multifactorial and are associated with complex interactions among parasites, pathogens, pesticide exposure, nutritional stress, habitat degradation, and climate-related environmental pressures [4,5,7,8]. Recent studies further suggest that these stressors often act synergistically, increasing colony susceptibility to disease and mortality [5,7,8]. *Varroa destructor* mites are considered the most significant biotic threat to honey bee health. These ectoparasites not only weaken individual bees through hemolymph feeding but also serve as vectors for a range of debilitating viruses, including deformed wing virus (DWV), thereby exacerbating colony mortality [9].

Addressing honey bee health threats remains a significant challenge for apicultural researchers and practitioners. Currently, there are no approved antiviral treatments for honey bee diseases, except HoneyGuard-R^®^ (Eagle Vet Tech Co., Seoul, Republic of Korea), making colony management, biosecurity, and vector control essential components of disease prevention strategies [10,11]. Antibiotics are commonly used to manage bacterial and fungal infections; however, their overuse has raised concerns about antibiotic residues in hive products and the development of drug-resistant pathogens [12]. Similarly, miticides used against *Varroa* mites are becoming less effective due to the emergence of resistant mite populations [13,14,15,16,17,18]. In light of these challenges, RNA interference (RNAi) has emerged as a promising and targeted biotechnological tool for managing honey bee diseases. RNAi offers a species-specific mechanism to silence the expression of critical pathogen and parasite genes, potentially enabling safer, more sustainable control methods. This review explores the potential benefits and limitations of RNAi technologies in enhancing honey bee health and mitigating colony losses.

## 2. Mechanism of RNAi in Honey Bee

**Figure 1:**
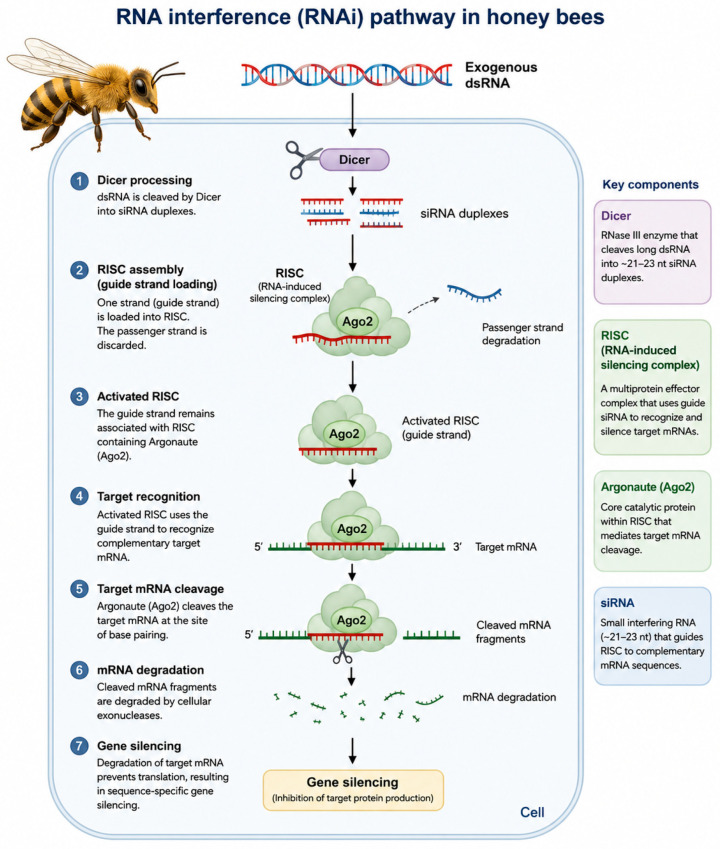
Overview of the RNA interference (RNAi) pathway in honey bees. Exogenous dsRNA is processed by Dicer into siRNAs. A guide siRNA strand (red color) is incorporated into the RNA-induced silencing complex (RISC), the effector complex responsible for RNAi-mediated gene silencing. RISC uses Argonaute proteins to recognize and cleave complementary target mRNAs, thereby preventing gene expression.

RNAi is a powerful, targeted gene-silencing mechanism with significant applications in insect research and pest management [19]. RNAi in insects begins with the introduction of exogenous double-stranded RNA (dsRNA), which can be delivered through microinjection, oral ingestion, or transgenic expression in plants for pest control [20]. Once inside the insect, dsRNA is taken up by cells via endocytosis or SID-like channels, depending on the species [21]. Inside the cell, the enzyme DICER processes the dsRNA into short fragments known as small interfering RNAs (siRNAs), typically 21~23 nucleotides in length [22]. These siRNAs are then incorporated into the RNA-Induced Silencing Complex (RISC), which guides the complex to a complementary messenger RNA (mRNA) target. Upon binding, the Argonaute (AGO) protein within RISC cleaves the mRNA, preventing its translation and thereby silencing the gene by reducing or eliminating the production of the corresponding protein [23] (Figure 1).

Although the core RNAi pathway described above is highly conserved across insects and many other eukaryotes, several features of RNAi efficiency and systemic spread vary among species [24,25]. Honey bees possess the essential RNAi machinery, including Dicer and Argonaute proteins, and utilize the canonical siRNA pathway in a manner broadly similar to that reported in other insects [26,27]. However, the uptake, persistence, and systemic distribution of dsRNA in honey bees may differ from those observed in model insects such as *Tribolium castaneum* or agricultural pests, contributing to variation in RNAi efficacy among species [24,28]. Therefore, while the molecular mechanism of RNAi is not unique to honey bees, understanding species-specific aspects of dsRNA delivery and gene silencing is critical for developing effective RNAi-based applications in apiculture [28].

RNAi has emerged as a valuable tool in honey bee research and pathogen control, offering a targeted approach to enhance bee health. Researchers have used RNAi to investigate gene function in honey bees, providing insights into developmental processes, immune responses, and behavior [29]. More importantly, RNAi is being explored as a strategy to combat viral infections that pose significant threats to honey bee populations. For instance, dsRNA targeting viral genes has been successfully used to reduce the replication and pathogenicity of the Israeli acute paralysis virus (IAPV), thereby improving honey bee survival rates [30]. Additionally, RNAi has shown potential in controlling other viruses such as deformed wing virus (DWV), which is often associated with *Varroa destructor* mite infestations [31]. These findings highlight RNAi as a promising method not only for functional genomics in honey bees but also for developing antiviral therapeutics to mitigate colony losses.

Experimental RNAi studies in honey bees have employed diverse dsRNA delivery strategies, including thoracic injection, oral administration via sugar syrup or pollen supplements, and more recently, symbiont-mediated delivery using engineered gut bacteria [28,30,32]. Injection-based approaches generally achieve rapid and efficient gene silencing under laboratory conditions but are impractical for colony-level implementation. In contrast, oral delivery methods are more compatible with commercial beekeeping practices, although their efficacy may vary depending on dsRNA stability, dosage, colony demographics, and feeding behavior [28]. Across different pathogens and target genes, RNAi treatments have produced substantial reductions in viral loads and target transcript abundance, with reported knockdown efficiencies typically ranging from 40% to 90% [30,32,33,34].

## 3. Utilizing RNAi for the Treatment of Viral Infections in Honey Bees

Viral pathogens pose a significant threat to honey bee health, contributing to reduced colony productivity and, in severe cases, colony collapse. Viruses such as DWV and IAPV have been linked to weakening bee colonies, leading to diminished honey yields, reduced brood production, and a decline in adult bee populations [35,36,37]. DWV, in particular, is associated with colony collapse disorder, in which entire colonies may die off or abandon their hives [38]. Additionally, viral infections increase honey bees’ susceptibility to other stressors, including parasites, pesticides, and poor nutrition, further compromising colony health [36,39]. The spread of viruses is not limited to managed bee populations; many honey bee viruses can also infect wild bee species, potentially contributing to declines in global pollinator populations [40]. Compounding this issue, the detection and management of viral infections are difficult, as many viruses can persist asymptomatically, making their impact hard to detect [7,35,40]. The *Varroa* mite, an invasive pest, plays a crucial role in the spread of these viruses, as it weakens bee immune systems and acts as a vector for many viral diseases [41,42]. The combination of viral infections and other environmental stressors has led to high annual losses of honey bee colonies, which have averaged 30~40% in the U.S. since 2006 [36,39]. With at least 20 known viruses affecting honey bees, including DWV, IAPV, and black queen cell virus (BQCV), the impact of viral pathogens is substantial, with effects ranging from paralysis and deformities to colony collapse [31,35,40]. As honey bees play a critical role in pollinating about one-third of the world’s food crops, addressing viral threats is essential for sustaining agricultural productivity and ecosystem health [43].

Cultural management practices are fundamental components of integrated pest management (IPM) strategies for controlling viral diseases in honey bees. These practices help alleviate colony stressors that can exacerbate viral infections, as viruses typically persist at low levels within colonies and become more prominent under stressful conditions [36]. Key elements of cultural management include promoting strong colony health through practices such as effective queen management, routine equipment rotation, and control of viral vectors such as *Varroa* mites [36,42,43]. *Varroa* mites not only weaken bees but also serve as vectors for several bee viruses, including DWV, which complicates viral transmission [44,45,46,47]. Regular monitoring of colony health, with particular attention to viral symptoms and screening for other pathogens such as *Nosema*, is crucial for early detection and management [3,39]. Although no chemotherapies are available to treat viral diseases in honey bees, effective management of these stressors can substantially reduce the risk of viral outbreaks and enhance colony resilience [41]. Furthermore, proper management of colonies under stress can prevent asymptomatic viral infections from progressing to more severe, symptomatic stages, making cultural management an essential tool for minimizing the impact of viral pathogens on bee populations [38].

RNAi serves as a major antiviral defense mechanism in honey bees through the production of virus-derived small interfering RNAs (siRNAs) that guide the degradation of viral RNA. Experimental studies have demonstrated activation of the RNAi pathway during viral infection and shown that disruption of key RNAi components compromises antiviral defense, resulting in increased viral replication and mortality [48,49,50]. Moreover, administration of virus-specific dsRNA has successfully reduced viral loads and improved bee survival in both laboratory and field studies, including those targeting IAPV, DWV, and SBV [30,33,51,52].

Several studies have demonstrated the effectiveness of RNAi-based approaches for suppressing honey bee viral infections. RNAi, a key antiviral defense mechanism in honey bees, involves the recognition and degradation of viral RNA by small interfering RNAs (siRNAs) generated via the RNAi pathway. Research has shown that honey bee colonies affected by colony collapse disorder (CCD) exhibit a strong RNAi response against several major honey bee viruses, such as DWV, IAPV, and Kashmir bee virus (KBV), indicating that RNAi is an effective mechanism for virus recognition and silencing [5,29,33]. Furthermore, the application of dsRNA has shown potential to reduce viral load, particularly for controlling sacbrood virus (SBV) in honey bee colonies, suggesting that dsRNA treatments could be scaled up for broader use [52]. One advantage of RNAi is its ability to target specific viral genes, providing highly targeted therapy with minimal off-target effects.

Additionally, RNAi can be administered through various delivery methods, including feeding or injection of siRNA, or using dsRNA products like Remebee-IAPV and HoneyGuard-R^®^, which have shown effectiveness in reducing virus-related mortality and improving colony health [5,51,52]. However, several challenges continue to limit the widespread application of RNAi-based antiviral therapies in honey bees. Viruses may evolve mechanisms to suppress the RNAi pathway, and efficient delivery of dsRNA to all bee tissues remains difficult [28]. In addition, the duration of the protective effect and the optimal frequency of dsRNA administration are not yet fully understood [51,53]. Beyond these biological constraints, large-scale implementation is further hindered by production costs, dsRNA instability under environmental conditions, and the difficulty of achieving effective colony-wide delivery. Commercial beekeeping operations require treatments that remain effective across diverse environmental conditions, colony sizes, and management systems while maintaining economic feasibility. Furthermore, repeated dsRNA administration may increase operational costs and limit scalability, particularly in large commercial and migratory beekeeping operations [54]. Nevertheless, advances in dsRNA formulation and delivery technologies, including nanoparticle-based carriers and symbiont-mediated RNAi platforms, have the potential to enhance dsRNA stability, improve delivery efficiency, and increase the practicality and cost-effectiveness of RNAi deployment under field conditions [55,56,57]. Despite these challenges, RNAi remains a promising tool for controlling viral diseases and improving honey bee health, with continued research focused on overcoming current limitations and facilitating commercial-scale application [28,34,48].

## 4. RNAi for Nosema Disease Treatment

*Nosema* infection, especially caused by *Nosema ceranae*, poses a serious threat to honey bee health and colony sustainability. Infected colonies often exhibit reduced adult bee populations, decreased brood rearing, and lower honey yields, directly impacting productivity [58,59]. At the individual level, infected worker bees may begin foraging prematurely, experience shortened lifespans, and suffer from impaired nursing abilities, which further destabilize colony dynamics [60]. In queens, *Nosema* infection can be fatal, leading to a cessation of egg-laying and death within a matter of weeks [61]. Additionally, *Nosema* increases colony vulnerability to collapse and winter mortality, compounding the risks to long-term colony survival [60,61]. The parasite invades and damages the midgut epithelial cells of honey bees, hindering digestion and nutrient absorption, while also inducing energetic stress, immune suppression, and behavioral changes [62]. Its spores are highly transmissible, allowing the disease to rapidly spread throughout a colony [61]. Given its significant implications for both managed and wild bee populations, *Nosema* infection is a global concern with far-reaching consequences for agricultural productivity and food security, as it reduces pollination services. Thus, vigilant monitoring and strategic management are essential for mitigating its impact.

Although fumagillin has been the principal treatment for *Nosema* infections in honey bees for decades, concerns regarding toxicity, residue accumulation in hive products, regulatory restrictions, and declining efficacy against *Nosema ceranae* have highlighted the need for safer and more sustainable alternatives [62,63,64,65]. Recent studies have evaluated RNAi-based approaches for suppressing *Nosema ceranae* infections in honey bees. One notable approach involves silencing mitosome-related genes in *N. ceranae*, such as NCER_101456 and NCER_100157, using dsRNA. This method significantly reduced spore production and improved the survival of infected bees, demonstrating RNAi’s potential to suppress vital parasite functions [65,66]. Another RNAi-based technique targets the parasite’s structural integrity by silencing genes encoding spore wall proteins (SWP8 and SWP12), thereby decreasing infection levels, enhancing immunity, and extending the lifespan of treated bees [67]. Beyond direct RNA delivery, researchers have also leveraged symbiont-mediated RNAi by engineering the honey bee gut bacterium *Snodgrassella alvi* to express dsRNA targeting essential *N. ceranae* genes. This approach effectively reduced *Nosema* proliferation and improved bee survival, even among older forager bees [56]. Notably, the engineered bacteria were shown to transmit between cohoused bees, enabling colony-level protection and making this method a scalable solution for *Nosema* management [56]. Similarly, Lang et al. (2023) [68] demonstrated that interfering with the *Nosema* redox system using engineered symbiotic bacteria could enhance host resistance to infection. Together, these studies underscore the transformative potential of RNAi-based technologies—particularly those using microbial symbionts—as sustainable, targeted alternatives to conventional chemical treatments that *Nosema* has increasingly become resistant to.

Despite the promising potential of RNAi in controlling *Nosema ceranae* infections in honey bees, several vital limitations remain that hinder its practical application. One key issue is the inconsistent efficacy of RNAi treatments. While some studies have demonstrated that dsRNA targeting specific *Nosema* genes can reduce spore loads and improve bee survival, the effectiveness varies significantly depending on the gene target, with some dsRNAs showing limited inhibition of spore proliferation [55,65,68]. Furthermore, combining multiple dsRNAs targeting different genes has not reliably produced synergistic effects, suggesting that further research is needed to identify optimal gene targets and combinations [66]. Delivery remains another major hurdle; although approaches such as nanocarriers have been proposed to enhance dsRNA stability and uptake, the most effective and scalable delivery method in field conditions is still under investigation [68].

Additionally, while RNAi is considered relatively specific, the potential for off-target effects—either in honey bees or in non-target environmental organisms—requires careful risk assessment. Perhaps most critically, the majority of RNAi studies have been conducted in controlled laboratory settings, and their real-world effectiveness in large-scale apiculture has yet to be thoroughly validated. As such, although RNAi remains a compelling candidate for *Nosema* management, further research is essential to overcome these technical and ecological challenges before widespread field application becomes viable.

## 5. RNAi for Varroa Mite Control

The *Varroa destructor* mite poses one of the most significant threats to honey bee health and global apiculture. Widely recognized as a primary driver of honey bee decline, especially in *Apis mellifera* populations that lack natural resistance, *Varroa* mites have contributed to severe colony losses worldwide [45,47]. These mites feed parasitically on the fat body tissue and hemolymph of both developing brood and adult bees, leading to weakened individuals with shortened lifespans, compromised immune systems, and impaired physiological functions [46]. In addition to their direct effects, *Varroa* mites serve as efficient vectors of devastating honey bee viruses, such as DWV, IAPV, and BQCV, which exacerbate colony morbidity and mortality [47]. Colonies suffering from heavy infestations display scattered brood patterns, deformed bees, impaired flight ability, and reduced worker bee weight and longevity—symptoms that often culminate in colony collapse. Alarmingly, *Varroa* mite populations can expand rapidly within a few years if not managed, overwhelming bee colonies’ natural defenses [46,69,70]. Given its widespread prevalence and multifaceted impact, the *Varroa* mite is regarded as a leading cause of honey bee losses and a critical concern for beekeepers and researchers alike.

*Varroa* mite control relies heavily on the integrated pest management (IPM) strategies that combine cultural, mechanical, chemical, and biological approaches to limit infestation while minimizing harm to bees and the environment. Cultural methods, such as creating brood breaks and selectively breeding mite-resistant honey bee strains, aim to disrupt the mite reproductive cycle and promote long-term colony resilience [71]. Mechanical interventions, such as screened bottom boards and sugar dusting, help dislodge mites from bees, offering non-chemical control options [72]. Chemical treatments are often divided into natural compounds—including thymol, formic acid, oxalic acid, and hop beta acids—which can be effective when applied correctly but may vary in efficacy depending on environmental conditions [72,73,74]. Synthetic miticides, such as amitraz, tau-fluvalinate, and coumaphos, have traditionally provided strong mite suppression; however, widespread and repeated use has led to the development of mite resistance, reducing their effectiveness and raising concerns about residues in hive products and potential harm to bees [14,16,18]. Despite the variety of available methods, each has limitations, and overreliance on any single approach can lead to resistance or reduced efficacy, underscoring the need for rotation and combination within a comprehensive IPM framework [71,75].

RNAi has emerged as a promising species-specific approach for controlling *Varroa destructor*, one of the most damaging parasites of honey bees. Early studies demonstrated that gene expression in *V. destructor* could be successfully suppressed through dsRNA-mediated RNAi, providing the first evidence that RNAi could be exploited for mite control [76]. Subsequently, Garbian et al. [77] showed that dsRNA administered to honey bees could be transferred to feeding mites, resulting in silencing of essential mite genes and significant reductions in mite populations. These findings established the feasibility of host-mediated RNAi as a novel strategy for *Varroa* management.

Since then, several studies have targeted genes involved in mite survival, development, and reproduction. RNAi-mediated silencing of genes such as vitellogenin, actin, calmodulin, and aquaporins has been associated with reduced mite fecundity, impaired physiological functions, and decreased population growth [78]. More recently, McGruddy et al. [78] demonstrated that dsRNA targeting reproduction-associated genes significantly suppressed mite reproduction under colony conditions. In parallel, engineered symbiont-based delivery systems have emerged as an innovative approach for colony-wide RNAi deployment. Leonard et al. [32] demonstrated that genetically modified *Snodgrassella alvi* could continuously produce dsRNA within the honey bee gut, inducing RNAi responses in both bees and parasitic mites and providing sustained suppression of *Varroa* populations.

For practical application, dsRNA can be delivered through feeding, allowing mites to acquire RNA molecules while parasitizing brood or adult bees [77,78]. Compared with conventional acaricides, RNAi offers high target specificity and reduces concerns regarding chemical residues and resistance development [32,70]. Nevertheless, several challenges remain, including the need for repeated applications, optimization of delivery systems, and validation of long-term efficacy under commercial beekeeping conditions [76,78]. Encouragingly, recent field-scale evaluations have reported substantial reductions in mite infestation levels following RNAi treatment, supporting its potential integration into future IPM programs for *Varroa destructor* control [79].

The recent commercialization of RNAi-based technologies further highlights the practical potential of this approach for *Varroa* management. In 2025, the United States Environmental Protection Agency (EPA) approved Norroa™ (vadescana), developed by GreenLight Biosciences (now Vadescana), as the first RNAi-based biopesticide for controlling *Varroa destructor* in honey bee colonies [80]. The product contains double-stranded RNA targeting the *Varroa* calmodulin gene, which is silenced through RNA interference following ingestion by the mite, resulting in reduced reproduction and survival while maintaining high specificity toward the target pest [81,82]. Experimental studies demonstrated that vadescana suppresses calmodulin expression and disrupts embryo development pathways, thereby reducing mite reproductive success [81]. Furthermore, field evaluations conducted under commercial beekeeping conditions reported significant reductions in *Varroa* infestation levels, supporting the feasibility of RNAi as a commercially deployable tool within integrated pest management (IPM) programs [83]. The approval of Norroa™ marks a major milestone in the development of RNAi-based pest control technologies and provides the first commercial proof-of-concept for RNAi-mediated *Varroa* management [80,83]. Nevertheless, further studies are required to evaluate long-term efficacy, cost-effectiveness, resistance risk, environmental persistence, and integration with existing IPM strategies under diverse beekeeping conditions [84].

Additionally, although RNAi does not alter the DNA of the species, further research is needed to understand its long-term effects on non-target species [78,85]. Studies have shown that RNAi can significantly reduce mite populations, with some research indicating reductions of over 60% [67,86]. Future research is focused on optimizing the dsRNA sequences to target more vital genes, improving the efficiency of delivery systems, and monitoring long-term impacts in field conditions [78,79,85].

## 6. RNAi for Small Hive Beetle Control

The small hive beetle (SHB), *Aethina tumida* Murray, is an invasive pest of honey bee colonies that has expanded from its native range in sub-Saharan Africa to multiple regions worldwide, where it can cause substantial economic losses to apiculture [68,69]. Comprehensive reviews have highlighted SHB as a global biosecurity concern due to its remarkable ecological flexibility, broad host associations, and increasing geographic distribution [87,88,89]. SHB larvae are the most destructive life stage, feeding on honey, pollen, and bee brood while tunneling through combs and contaminating hive products with feces and associated microorganisms, resulting in honey fermentation, comb destruction, and colony deterioration [87,88]. Under favorable environmental conditions, severe infestations can trigger colony absconding and significantly reduce colony productivity and honey yields [87,88]. The pest is particularly problematic in warm and humid climates that facilitate beetle reproduction and pupation in surrounding soil [90]. Consequently, SHB remains a major threat to apiculture and continues to be the focus of surveillance and management programs worldwide.

RNA interference (RNAi) has emerged as a promising species-specific approach for SHB management. The first evidence of systemic RNAi in *A. tumida* was reported by Powell et al. [91], who demonstrated that dsRNA targeting the essential genes V-ATPase subunit A and laccase 2 induced significant gene knockdown and resulted in complete mortality of injected larvae. Oral delivery of the same dsRNAs achieved lower efficacy, likely because of degradation within the digestive tract before sufficient cellular uptake could occur [91]. Importantly, the SHB-specific dsRNAs did not adversely affect honey bee survival or gene expression, highlighting the selectivity and potential environmental safety of RNAi-based pest control [91].

More recently, RNAi has also been applied as a functional genomics tool in SHB. Li et al. (2024) [92] demonstrated that silencing three odorant-binding protein genes (AtumOBP6, AtumOBP11, and AtumOBP19) significantly altered the beetle’s response to key honey bee colony volatiles, confirming that SHB is highly susceptible to gene silencing and revealing additional molecular targets for future RNAi-based control strategies [92]. These findings extend the potential application of RNAi beyond mortality-based approaches toward behavioral disruption and host-finding interference.

Despite these encouraging laboratory results, RNAi-based SHB control remains at an early stage of development. Major challenges include improving oral delivery efficiency, protecting dsRNA from nuclease degradation, reducing production costs, and validating efficacy under colony-level and field conditions. Continued advances in dsRNA stabilization technologies, nanoparticle-assisted delivery systems, and RNA-based biopesticide platforms may facilitate the translation of laboratory findings into practical SHB management tools in commercial beekeeping operations.

## 7. Engineered Endosymbionts Producing RNAi Offer a Promising Method for Controlling Pathogens and Parasites in Honey Bees

Engineered endosymbionts producing RNAi represent a promising and innovative strategy for preventing and treating honey bee pathogens and parasites, addressing key challenges such as delivery efficiency and cost in field applications. This approach utilizes genetically modified gut bacteria—specifically *Snodgrassella alvi*, a natural symbiont of honey bees—to produce dsRNA that activates the RNAi pathway. Known as Functional Genomics Using Engineered Symbionts (FUGUES), this method enables persistent, systemic knockdown of targeted genes in honey bees by up to 75% [54]. When engineered *S. alvi* colonizes bees, the symbionts not only improve survival after viral infections but also induce RNAi in *Varroa destructor*, significantly reducing mite populations [54]. This dual-action effect offers a sustainable alternative to traditional treatments, particularly against viral threats like DWV, which are exacerbated by mite infestations [86]. Beyond treatment, symbiont-mediated RNAi also provides a powerful tool for functional genomics in honey bees, facilitating gene-phenotype studies that were previously difficult to perform. Overall, this method holds great promise for enhancing honey bee resilience through precise, eco-friendly, and scalable biotechnological solutions.

**Figure 2:**
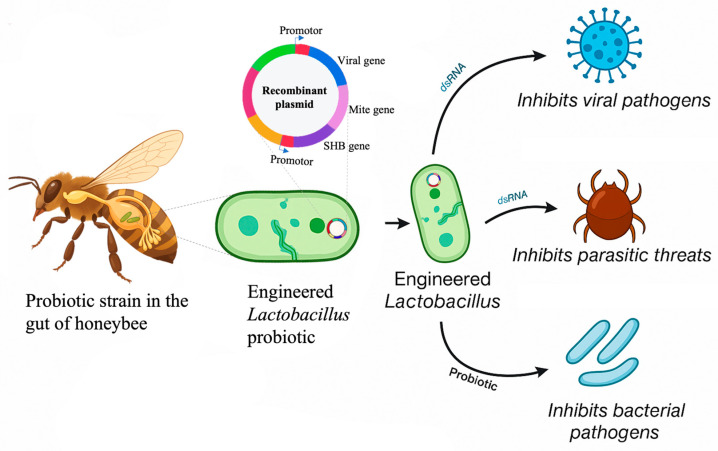
Conceptual illustration of engineered *Lactobacillus* symbionts producing dsRNA to suppress honey bee pathogens and parasites while supporting gut health.

Using probiotic *Lactobacillus* strains to engineer honey bee endosymbionts that carry recombinant plasmids encoding dsRNA targeting species-specific genes of viral pathogens and parasites offers a promising strategy for enhancing honey bee health and resilience. *Lactobacillus* species, such as *Lactobacillus apis* and *Lactobacillus kunkeei*, are naturally present in the honey bee gut and play a vital role in maintaining gut health, modulating immune responses, and protecting against bacterial pathogens, such as *Paenibacillus larvae*, the causative agent of American foulbrood [32,93]. These beneficial bacteria can be engineered to produce dsRNA that inhibits viral pathogens, such as DWV, and parasitic threats, such as *Varroa* mites, thereby directly targeting the pathogens that affect bee colonies. The expression of dsRNA can be controlled by using promoters from *Lactobacillus* species, such as the constitutive *Pldh*L promoter from *Lactobacillus plantarum* or the inducible *Pnis*A promoter from *Lactococcus lactis* [94,95,96]. Additionally, engineered *Lactobacillus* strains can enhance bees’ immune function by supporting a balanced gut microbiome, which is critical for their ability to resist infections and environmental stressors [97,98,99]. This dual functionality of engineered *Lactobacillus* strains, as both a probiotic and a delivery system for RNAi-based pathogen control, provides a sustainable, targeted approach to protecting honey bees from the increasing challenges posed by pathogens and environmental changes (Figure 2).

## 8. Challenges and Future Perspectives

Although RNAi-based technologies offer considerable promise for sustainable honey bee health management, regulatory approval and environmental risk assessment remain important prerequisites for commercial deployment. Because honey bees are directly linked to food production systems and ecosystem services, RNAi-based products must satisfy rigorous regulatory requirements regarding human safety, environmental fate, residue persistence, and impacts on non-target organisms [100,101,102,103,104]. Recent regulatory frameworks developed by international organizations, including the OECD and EFSA, emphasize the need for case-specific assessments of dsRNA exposure, degradation, and ecological safety before field application can be broadly adopted [100,105].

Another important consideration is the potential evolution of resistance. Similar to conventional pesticides, prolonged exposure to a single RNAi target may select for resistance mechanisms, including sequence variation within target genes, reduced cellular uptake of dsRNA, altered RNAi machinery, or increased nuclease-mediated degradation of dsRNA molecules [24,106]. Consequently, RNAi-based interventions should be integrated into broader IPM programs incorporating treatment rotation and complementary control measures to minimize selection pressure and prolong efficacy.

Environmental persistence and off-target effects also warrant continued investigation. Available evidence suggests that dsRNA generally degrades relatively rapidly in environmental matrices; however, its persistence within hive-associated materials such as wax, pollen, honey, and hive debris under field conditions remains insufficiently characterized [101,102,103,104,107]. Furthermore, although RNAi is considered highly sequence-specific, comprehensive assessments of potential effects on beneficial arthropods, native pollinators, and microbial communities remain necessary to support long-term environmental safety [100,102,103,104].

Finally, cost-effective dsRNA production, formulation stability, and practical colony-level delivery remain critical challenges for large-scale implementation. Continued advances in microbial fermentation, cell-free RNA synthesis, nanoparticle-based stabilization, and symbiont-mediated delivery systems are expected to improve the economic feasibility and durability of RNAi applications in apiculture. Achieving successful commercialization will require close collaboration among molecular biologists, ecologists, regulatory agencies, and the beekeeping industry to translate promising laboratory findings into reliable field-ready technologies [56,57,108,109].

## 9. Conclusions

RNAi represents a promising and highly selective technology for controlling major honey bee pathogens and pests, including viruses, *Nosema ceranae*, *Varroa destructor*, and the small hive beetle (*Aethina tumida*) (Table 1). Current evidence demonstrates that dsRNA-mediated gene silencing can reduce pathogen replication, impair parasite development and reproduction, and improve honey bee survival. While antiviral and symbiont-mediated RNAi approaches have shown the greatest potential for colony-level application, RNAi-based control of *Varroa* and SHB remains under active development. Together with ongoing advances in delivery systems and formulation technologies, RNAi has strong potential to become an important component of future IPM strategies aimed at improving honey bee health and colony sustainability.

## Figures and Tables

**Table 1:** RNAi applications in honey bee disease and pest management.

Target	Target Genes	Delivery Method	Scale Applicability	Effect/Outcome	References
Viral pathogens	DWV, IAPV, SBV, KBV genes	Injection, feeding, engineered symbionts	Laboratory to semi-field; Colony-level applicable	Reduced viral load, increased survival	[30,33,34,53,54,58]
*Nosema* *ceranae*	SWP8, SWP12, Mitosome-related genes, Redox system	Oral dsRNA, engineered symbionts	Potential colony-level use	Reduced spore load, improved immunity	[56,66]
*Varroa* *destructor*	Vitellogenin, aquaporins, apoptosis- related genes	Feeding through bees, engineered symbionts	Semi-field and field tested	Reduced mite fertility and infestation	[32,54,78,86]
Small Hive Beetle	V-ATPase, laccase 2	Injection, oral feeding	Primarily laboratory stage	High larval mortality	[91,110]

## Data Availability

No new data were created or analyzed in this study. Data sharing is not applicable to this article.

## Abbreviations

The following abbreviations are used in this manuscript:

RNAi	RNA interference
dsRNA	double-stranded RNA
DWV	Deformed Wing Virus
SHB	Small hive beetles
RISC	RNA-Induced Silencing Complex
mRNA	Messenger RNA
IAPV	Israeli Acute Paralysis Virus
BQCV	Black Queen Cell Virus
siRNAs	Small interfering RNAs
*N. ceranae*	*Nosema ceranae*
qPCR	Quantitative Polymerase Chain Reaction

## References

[1] Hung K.-L.J., Kingston J.M., Albrecht M., Holway D.A., Kohn J.R. (2018). The Worldwide Importance of Honey Bees as Pollinators in Natural Habitats. Proc. R. Soc. B Biol. Sci..

[2] DeGrandi-Hoffman G., Chen Y., Simonds R. (2013). The Effects of Pesticides on Queen Rearing and Virus Titers in Honey Bees (*Apis mellifera* L.). Insects.

[3] Kulhanek K., Steinhauer N., Rennich K., Caron D.M., Sagili R.R., Pettis J.S., Ellis J.D., Wilson M.E., Wilkes J.T., Tarpy D.R. (2017). A National Survey of Managed Honey Bee 2015–2016 Annual Colony Losses in the USA. J. Apic. Res..

[4] Goulson D., Nicholls E., Botias C., Rotheray E.L. (2015). Bee Declines Driven by Combined Stress from Parasites, Pesticides, and Lack of Flowers. Science.

[5] Warner S., Pokhrel L.R., Akula S.M., Ubah C.S., Richards S.L., Jensen H., Kearney G.D. (2024). A Scoping Review on the Effects of *Varroa* Mite (*Varroa destructor*) on Global Honey Bee Decline. Sci. Total Environ..

[6] Nearman A., Crawford C.L., Guarna M.M., Chakrabarti P., Lee K., Cook S., Hill E., Seshadri A., Slater G., Lamas Z.S. (2025). Insights from U.S. Beekeeper Triage Surveys Following Unusually High Honey Bee Colony Losses 2024–2025. Sci. Total Environ..

[7] McMenamin A.J., Genersch E. (2015). Honey Bee Colony Losses and Associated Viruses. Curr. Opin. Insect Sci..

[8] Insolia L., Molinari R., Rogers S.R., Williams G.R., Chiaromonte F., Calovi M. (2022). Honey Bee Colony Loss Linked to Parasites, Pesticides and Extreme Weather across the United States. Sci. Rep..

[9] Rosenkranz P., Aumeier P., Ziegelmann B. (2010). Biology and Control of *Varroa destructor*. J. Invertebr. Pathol..

[10] Brutscher L.M., Flenniken M.L. (2015). RNAi and Antiviral Defense in the Honey bee. J. Immunol..

[11] Brutscher L.M., Daughenbaugh K.F., Flenniken M.L. (2015). Antiviral Defense Mechanisms in Honey Bees. Curr. Opin. Insect Sci..

[12] Reybroeck W. (2017). Residues of Antibiotics and Chemotherapeutics in Honey. J. Apic. Res..

[13] Elzen P.J., Westervelt D., Lucas R. (2004). Formic Acid Treatment for Control of *Varroa destructor* (Mesostigmata: Varroidae) and Safety to *Apis mellifera* (Hymenoptera: Apidae) under Southern United States Conditions. J. Econ. Entomol..

[14] Hernández-Rodríguez C.S., Marín Ó., Calatayud F., Mahiques M.J., Mompó A., Segura I., Simó E., González-Cabrera J. (2021). Large-Scale Monitoring of Resistance to Coumaphos, Amitraz, and Pyrethroids in *Varroa destructor*. Insects.

[15] González-Cabrera J., Davies T.G.E., Field L.M., Kennedy P.J., Williamson M.S. (2013). An Amino Acid Substitution (L925V) Associated with Resistance to Pyrethroids in *Varroa destructor*. PLoS ONE.

[16] Maggi M.D., Ruffinengo S.R., Negri P., Eguaras M.J. (2010). Resistance Phenomena to Amitraz from Populations of the Ectoparasitic Mite *Varroa destructor* of Argentina. Parasitol. Res..

[17] Hernández-Rodríguez C.S., Moreno-Martí S., Emilova-Kirilova K., González-Cabrera J. (2025). A New Mutation in the Octopamine Receptor Associated with Amitraz Resistance in *Varroa destructor*. Pest Manag. Sci..

[18] Millán-Leiva A., Marín Ó., Christmon K., vanEngelsdorp D., González-Cabrera J. (2021). Mutations Associated with Pyrethroid Resistance in *Varroa* Mite, a Parasite of Honey Bees, Are Widespread across the United States. Pest Manag. Sci..

[19] Whyard S., Singh A.D., Wong S. (2009). Ingested Double-Stranded RNAs Can Act as Species-Specific Insecticides. Insect Biochem. Mol. Biol..

[20] Baum J.A., Bogaert T., Clinton W., Heck G.R., Feldmann P., Ilagan O., Johnson S., Plaetinck G., Munyikwa T., Pleau M. (2007). Control of Coleopteran Insect Pests through RNA Interference. Nat. Biotechnol..

[21] Huvenne H., Smagghe G. (2010). Mechanisms of DsRNA Uptake in Insects and Potential of RNAi for Pest Control: A Review. J. Insect Physiol..

[22] Fire A., Xu S., Montgomery M.K., Kostas S.A., Driver S.E., Mello C.C. (1998). Potent and Specific Genetic Interference by Double-Stranded RNA in Caenorhabditis Elegans. Nature.

[23] Hammond S.M., Bernstein E., Beach D., Hannon G.J. (2000). An RNA-Directed Nuclease Mediates Post-Transcriptional Gene Silencing in Drosophila Cells. Nature.

[24] Cooper A.M., Silver K., Zhang J., Park Y., Zhu K.Y. (2019). Molecular Mechanisms Influencing Efficiency of RNA Interference in Insects. Pest Manag. Sci..

[25] Joga M.R., Zotti M.J., Smagghe G., Christiaens O. (2016). RNAi Efficiency, Systemic Properties, and Novel Delivery Methods for Pest Insect Control: What We Know So Far. Front. Physiol..

[26] Evans J.D., Aronstein K., Chen Y.P., Hetru C., Imler J.-L., Jiang H., Kanost M., Thompson G.J., Zou Z., Hultmark D. (2006). Immune Pathways and Defence Mechanisms in Honey Bees *Apis mellifera*. Insect Mol. Biol..

[27] Maori E., Garbian Y., Kunik V., Mozes-Koch R., Malka O., Kalev H., Sabath N., Sela I., Shafir S. (2019). A Transmissible RNA Pathway in Honey Bees. Cell Rep..

[28] Yang D., Xu X., Zhao H., Yang S., Wang X., Zhao D., Diao Q., Hou C. (2018). Diverse Factors Affecting Efficiency of RNAi in Honey Bee Viruses. Front. Genet..

[29] Azzami K., Ritter W., Tautz J., Beier H. (2012). Infection of Honey Bees with Acute Bee Paralysis Virus Does Not Trigger Humoral or Cellular Immune Responses. Arch. Virol..

[30] Maori E., Paldi N., Shafir S., Kalev H., Tsur E., Glick E., Sela I. (2009). IAPV, a Bee-Affecting Virus Associated with Colony Collapse Disorder Can Be Silenced by DsRNA Ingestion. Insect Mol. Biol..

[31] Smeele Z.E., Baty J.W., Lester P.J. (2023). Effects of Deformed Wing Virus-Targeting DsRNA on Viral Loads in Bees Parasitised and Non-Parasitised by *Varroa destructor*. Viruses.

[32] Leonard S.P., Powell J.E., Perutka J., Geng P., Heckmann L.C., Horak R.D., Davies B.W., Ellington A.D., Barrick J.E., Moran N.A. (2020). Engineered Symbionts Activate Honey Bee Immunity and Limit Pathogens. Science.

[33] Hunter W., Ellis J., vanEngelsdorp D., Hayes J., Westervelt D., Glick E., Williams M., Sela I., Maori E., Pettis J. (2010). Large-Scale Field Application of RNAi Technology Reducing Israeli Acute Paralysis Virus Disease in Honey Bees (*Apis mellifera*, Hymenoptera: Apidae). PLoS Pathog..

[34] Ferrufino C., Scannapieco A., Russo R.M., Gonzalez F.N., Salvador R., Dus Santos M.J. (2025). Reduction in Acute Bee Paralysis Virus Infection and Mortality in Honey Bees (*Apis mellifera*) by RNA Interference Technology. Insects.

[35] Grozinger C.M., Flenniken M.L. (2019). Bee Viruses: Ecology, Pathogenicity, and Impacts. Annu. Rev. Entomol..

[36] Ullah A., Tlak Gajger I., Majoros A., Dar S.A., Khan S., Kalimullah, Haleem Shah A., Nasir Khabir M., Hussain R., Khan H.U. (2021). Viral Impacts on Honey Bee Populations: A Review. Saudi J. Biol. Sci..

[37] Woodford L., Evans D.J. (2021). Deformed Wing Virus: Using Reverse Genetics to Tackle Unanswered Questions about the Most Important Viral Pathogen of Honey Bees. FEMS Microbiol. Rev..

[38] Sumpter D.J.T., Martin S.J. (2004). The Dynamics of Virus Epidemics in *Varroa*-Infested Honey Bee Colonies. J. Anim. Ecol..

[39] vanEngelsdorp D., Evans J.D., Saegerman C., Mullin C., Haubruge E., Nguyen B.K., Frazier M., Frazier J., Cox-Foster D., Chen Y. (2009). Colony Collapse Disorder: A Descriptive Study. PLoS ONE.

[40] Dolezal A.G., Hendrix S.D., Scavo N.A., Carrillo-Tripp J., Harris M.A., Wheelock M.J., O’Neal M.E., Toth A.L. (2016). Honey Bee Viruses in Wild Bees: Viral Prevalence, Loads, and Experimental Inoculation. PLoS ONE.

[41] Tantillo G., Bottaro M., Di Pinto A., Martella V., Di Pinto P., Terio V. (2015). Virus Infections of Honeybees *Apis mellifera*. Ital. J. Food Saf..

[42] Truong A.-T., Yoo M.-S., Yun B.-R., Kang J.E., Noh J., Hwang T.J., Seo S.K., Yoon S.-S., Cho Y.S. (2023). Prevalence and Pathogen Detection of *Varroa* and *Tropilaelaps* Mites in *Apis mellifera* (Hymenoptera, Apidae) Apiaries in South Korea. J. Apic. Res..

[43] Gallai N., Salles J.M., Settele J., Vaissière B. (2009). Economic Valuation of the Vulnerability of World Agriculture Confronted with Pollinator Decline. Ecol. Econ..

[44] Chen Y.P., Siede R. (2007). Honey Bee Viruses. Adv. Virus Res..

[45] Paudel Y., Mackereth R., Hanley R., Qin W. (2015). Honey Bees (*Apis mellifera* L.) and Pollination Issues: Current Status, Impacts and Potential Drivers of Decline. J. Agric. Sci..

[46] Morfin N., Goodwin P.H., Guzman-Novoa E. (2023). *Varroa destructor* and Its Impacts on Honey Bee Biology. Front. Bee Sci..

[47] Martin S.J. (2001). The Role of *Varroa* and Viral Pathogens in the Collapse of Honeybee Colonies: A Modelling Approach. J. Appl. Ecol..

[48] Brutscher L.M., Daughenbaugh K.F., Flenniken M.L. (2017). Virus and DsRNA-Triggered Transcriptional Responses Reveal Key Components of Honey Bee Antiviral Defense. Sci. Rep..

[49] Galbraith D.A., Yang X., Niño E.L., Yi S., Grozinger C. (2015). Parallel Epigenomic and Transcriptomic Responses to Viral Infection in Honey Bees (*Apis mellifera*). PLoS Pathog..

[50] Flenniken M.L., Andino R. (2013). Non-Specific DsRNA-Mediated Antiviral Response in the Honey Bee. PLoS ONE.

[51] Desai S.D., Eu Y.-J., Whyard S., Currie R.W. (2012). Reduction in Deformed Wing Virus Infection in Larval and Adult Honey Bees (*Apis mellifera* L.) by Double-stranded RNA Ingestion. Insect Mol. Biol..

[52] Yoo M.S., Truong A.T., Jeong H., Hahn D.H., Lee J.S., Yoon S.S., Youn S.Y., Cho Y.S. (2023). Large-Scale Application of Double-Stranded RNA Shows Potential for Reduction of Sacbrood Virus Disease in *Apis cerana* Apiaries. Viruses.

[53] Zhang Y., Li Z., Wang Z.-L., Zhang L.-Z., Zeng Z.-J. (2022). A Comparison of RNA Interference via Injection and Feeding in Honey Bees. Insects.

[54] Lariviere P.J., Leonard S.P., Horak R.D., Powell J.E., Barrick J.E. (2023). Honey Bee Functional Genomics Using Symbiont-Mediated RNAi. Nat. Protoc..

[55] Qi Y., Wang C., Lang H., Wang Y., Wang X., Zheng H., Lu Y. (2024). Liposome-Based RNAi Delivery in Honeybee for Inhibiting Parasite *Nosema ceranae*. Synth. Syst. Biotechnol..

[56] Huang Q., Lariviere P.J., Powell J.E., Moran N.A. (2023). Engineered Gut Symbiont Inhibits Microsporidian Parasite and Improves Honey Bee Survival. Proc. Natl. Acad. Sci. USA.

[57] Quilez-Molina A.I., Niño Sanchez J., Merino D. (2024). The Role of Polymers in Enabling RNAi-Based Technology for Sustainable Pest Management. Nat. Commun..

[58] Zhu K.Y., Palli S.R. (2020). Mechanisms, Applications, and Challenges of Insect RNA Interference. Annu. Rev. Entomol..

[59] Botías C., Martín-Hernández R., Barrios L., Meana A., Higes M. (2013). *Nosema* spp. Infection and Its Negative Effects on Honey Bees (*Apis mellifera iberiensis*) at the Colony Level. Vet. Res..

[60] Paris L., Peghaire E., Moné A., Diogon M., Debroas D., Delbac F., El Alaoui H. (2020). Honeybee Gut Microbiota Dysbiosis in Pesticide/Parasite Co-Exposures Is Mainly Induced by *Nosema ceranae*. J. Invertebr. Pathol..

[61] Goblirsch M. (2018). *Nosema ceranae* Disease of the Honey Bee (*Apis mellifera*). Apidologie.

[62] Panek J., Paris L., Roriz D., Mone A., Dubuffet A., Delbac F., Diogon M., Alaoui H.E. (2018). Impact of the Microsporidian *Nosema ceranae* on the Gut Epithelium Renewal of the Honeybee, *Apis mellifera*. J. Invertebr. Pathol..

[63] Huang W.F., Solter L.F., Yau P.M., Imai B.S. (2013). *Nosema ceranae* Escapes Fumagillin Control in Honey Bees. PLoS Pathog..

[64] Higes M., Nozal M.J., Alvaro A., Barrios L., Meana A., Martín-Hernández R., Bernal J.L., Bernal J. (2011). The Stability and Effectiveness of Fumagillin in Controlling *Nosema ceranae* (Microsporidia) Infection in Honey Bees (*Apis mellifera*) under Laboratory and Field Conditions. Apidologie.

[65] Porrini M.P., Sarlo E.G., Medici S.K., Garrido P.M., Porrini D.P., Damiani N., Eguaras M.J. (2011). *Nosema ceranae* Development in *Apis mellifera*: Influence of Diet and Infective Inoculum. J. Apic. Res..

[66] Kim I.H., Kim D.J., Gwak W.S., Woo S.D. (2020). Increased Survival of the Honey Bee *Apis mellifera* Infected with the Microsporidian *Nosema ceranae* by Effective Gene Silencing. Arch. Insect Biochem. Physiol..

[67] He N., Zhang Y., Duan X.L., Li J.H., Huang W.F., Evans J.D., DeGrandi-Hoffman G., Chen Y.P., Huang S.K. (2021). RNA Interference-Mediated Knockdown of Genes Encoding Spore Wall Proteins Confers Protection against *Nosema ceranae* Infection in the European Honey Bee, *Apis mellifera*. Microorganisms.

[68] Lang H., Wang H., Wang H., Zhong Z., Xie X., Zhang W., Guo J., Meng L., Hu X., Zhang X. (2023). Engineered Symbiotic Bacteria Interfering *Nosema* Redox System Inhibit Microsporidia Parasitism in Honeybees. Nat. Commun..

[69] Traynor K.S., Mondet F., de Miranda J.R., Techer M., Kowallik V., Oddie M.A.Y., Chantawannakul P., McAfee A. (2020). *Varroa destructor*: A Complex Parasite, Crippling Honey Bees Worldwide. Trends Parasitol..

[70] Noël A., Le Conte Y., Mondet F. (2020). *Varroa destructor*: How Does It Harm *Apis mellifera* Honey Bees and What Can Be Done about It?. Emerg. Top. Life Sci..

[71] Ayan A., Tutun H., Aldemir O.S. (2019). Control Methods against *Varroa* Mites. Int. J. Adv. Study Res. Work.

[72] Jack C.J., Ellis J.D. (2021). Integrated Pest Management Control of *Varroa destructor* (Acari: Varroidae), the Most Damaging Pest of (*Apis mellifera* L. (Hymenoptera: Apidae)) Colonies. J. Insect Sci..

[73] Narciso L., Topini M., Ferraiuolo S., Ianiro G., Marianelli C. (2024). Effects of Natural Treatments on the *Varroa* Mite Infestation Levels and Overall Health of Honey Bee (*Apis mellifera*) Colonies. PLoS ONE.

[74] Calderón R.A., Ramírez M., Ramírez F., Villalobos E. (2014). Effectiveness of Formic Acid and Thymol in the Control of *Varroa destructor* in Africanized Honey Bee Colonies. Agron. Costarric..

[75] Lipiński Z., Szubstarski J. (2007). Resistance of *Varroa destructor* to Most Commonly Used Synthetic Acaricides. Pol. J. Vet. Sci..

[76] Campbell E.M., Budge G.E., Bowman A.S. (2010). Gene-Knockdown in the Honey Bee Mite *Varroa destructor* by a Non-Invasive Approach: Studies on a Glutathione S-Transferase. Parasites Vectors.

[77] Garbian Y., Maori E., Kalev H., Shafir S., Sela I. (2012). Bidirectional Transfer of RNAi between Honey Bee and *Varroa destructor*: *Varroa* Gene Silencing Reduces *Varroa* Population. PLoS Pathog..

[78] McGruddy R.A., Smeele Z.E., Manley B., Masucci J.D., Haywood J., Lester P.J. (2024). RNA Interference as a Next-Generation Control Method for Suppressing *Varroa destructor* Reproduction in Honey Bee (*Apis mellifera*) Hives. Pest Manag. Sci..

[79] Bortolin F., Rigato E., Perandin S., Granato A., Zulian L., Millino C., Pacchioni B., Mutinelli F., Fusco G. (2025). First Evidence of the Effectiveness of a Field Application of RNAi Technology in Reducing Infestation of the Mite *Varroa destructor* in the Western Honey Bee (*Apis mellifera*). Parasites Vectors.

[80] U.S. Environmental Protection Agency (EPA) EPA Provides New Tool to Protect Honey Bees, Ensuring Safe and Abundant Food Supply. https://www.epa.gov/pesticides/epa-provides-new-tool-protect-honey-bees-ensuring-safe-and-abundant-food-supply.

[81] Smeele Z.E., McGruddy R.A., Baty J.W., Manley B., Narva K., De Neef E., Gordon E.R., Devisetty U.K., Youngs K., Felden A. (2026). An RNA Interference Biopesticide Reduces Reproduction of the Honey Bee Parasite *Varroa destructor* by Down-regulating Embryo Development Pathways. Pest Manag. Sci..

[82] Merk J., Anastasi M., McGruddy R., Manley B., Felden A., Lester P.J. (2026). Longevity and Foraging Performance of Honey Bees Treated with an RNAi-Based *Varroa destructor* Biopesticide. Sci. Rep..

[83] Rawn D., Prouty C., Gautam A., Jamison M., Talton W., Youngs K., Narva K., Manley B., Jack C. (2026). Evaluating the New Product Norroa^TM^ against *Varroa destructor* in Managed Honey Bee (*Apis mellifera*) Colonies. Front. Insect Sci..

[84] Lester P., Bulgarella M., Manley B., Masucci J., McGruddy R., Merk J., Narva K.E., Palmer S., Mercier O.R., Smeele Z. (2026). NorroaTM as a DsRNA Biopesticide for *Varroa destructor*: Insights from New Zealand Research on Mode of Action, Field Efficacy, Non-target Effects, and Social Acceptance. Front. Insect Sci..

[85] Nekoei S., Rezvan M., Khamesipour F., Mayack C., Molento M.B., Revainera P.D. (2023). A Systematic Review of Honey Bee (*Apis mellifera*, Linnaeus, 1758) Infections and Available Treatment Options. Vet. Med. Sci..

[86] Niu J., Shen G., Christiaens O., Smagghe G., He L., Wang J. (2018). Beyond Insects: Current Status and Achievements of RNA Interference in Mite Pests and Future Perspectives. Pest Manag. Sci..

[87] Cuthbertson A.G.S., Wakefield M.E., Powell M.E., Marris G.A., Anderson H., Budge G.E., Brown M.A. (2013). The Small Hive Beetle *Aethina tumida*: A Review of Its Biology and Control Measures. Curr. Zool..

[88] Hood W.M. (2004). The Small Hive Beetle, *Aethina tumida*: A Review. Bee World.

[89] Neumann P., Pettis J.S., Schäfer M.O. (2016). Quo Vadis *Aethina tumida*? Biology and Control of Small Hive Beetles. Apidologie.

[90] Tutun H., Sekercİ Y., Sevin S. (2022). Future Effects of Small Hive Beetle, *Aethina tumida*, on Honey Bee Colony in Turkey Based on Temperature Factor Using a Mathematical Model. Eur. Zool. J..

[91] Powell M.E., Bradish H.M., Gatehouse J.A., Fitches E.C. (2017). Systemic RNAi in the Small Hive Beetle *Aethina tumida* Murray (Coleoptera: Nitidulidae), a Serious Pest of the European Honey Bee *Apis mellifera*. Pest Manag. Sci..

[92] Li L., Wu L., Xu Y., Liu F., Zhao H. (2024). Three Odorant-Binding Proteins of Small Hive Beetles, *Aethina tumida*, Participate in the Response of Bee Colony Volatiles. Int. J. Biol. Macromol..

[93] Chege M., Kinyua J., Paredes J.C. (2023). *Lactobacillus kunkeei* Impacts the Health of Honey Bees, *Apis mellifera scutellata*, and Protects the Bees against the Opportunistic Pathogen *Serratia marcescens*. Int. J. Trop. Insect Sci..

[94] Mojgani N., Bagheri M., Ashique S., Islam A., Moharrami M., Modirrousta H., Hussain A. (2025). Honeybee Defense Mechanisms: Role of Honeybee Gut Microbiota and Antimicrobial Peptides in Maintaining Colony Health and Preventing Diseases. Microb. Pathog..

[95] Peirotén Á., Landete J.M. (2020). Natural and Engineered Promoters for Gene Expression in Lactobacillus Species. Appl. Microbiol. Biotechnol..

[96] Heiss S., Hörmann A., Tauer C., Sonnleitner M., Egger E., Grabherr R., Heinl S. (2016). Evaluation of Novel Inducible Promoter/Repressor Systems for Recombinant Protein Expression in *Lactobacillus plantarum*. Microb. Cell Fact..

[97] Kazi T.A., Acharya A., Mukhopadhyay B.C., Mandal S., Arukha A.P., Nayak S., Biswas S.R. (2022). Plasmid-Based Gene Expression Systems for Lactic Acid Bacteria: A Review. Microorganisms.

[98] Daisley B.A., Pitek A.P., Chmiel J.A., Gibbons S., Chernyshova A.M., Al K.F., Faragalla K.M., Burton J.P., Thompson G.J., Reid G. (2020). *Lactobacillus* spp. Attenuate Antibiotic-Induced Immune and Microbiota Dysregulation in Honey Bees. Commun. Biol..

[99] Daisley B.A., Pitek A.P., Torres C., Lowery R., Adair B.A., Al K.F., Niño B., Burton J.P., Allen-Vercoe E., Thompson G.J. (2023). Delivery Mechanism Can Enhance Probiotic Activity against Honey Bee Pathogens. ISME J..

[100] OECD (2020). Considerations for the Environmental Risk Assessment of the Application of Sprayed or Externally Applied DsRNA-Based Pesticides.

[101] Romeis J., Widmer F. (2020). Assessing the Risks of Topically Applied DsRNA-Based Products to Non-Target Arthropods. Front. Plant Sci..

[102] Dubelman S., Fischer J., Zapata F., Huizinga K., Jiang C., Uffman J., Levine S., Carson D. (2014). Environmental Fate of Double-Stranded RNA in Agricultural Soils. PLoS ONE.

[103] Parker K.M., Barragán Borrero V., van Leeuwen D.M., Lever M.A., Mateescu B., Sander M. (2019). Environmental Fate of RNA Interference Pesticides: Adsorption and Degradation of Double-Stranded RNA Molecules in Agricultural Soils. Environ. Sci. Technol..

[104] Bachman P., Fischer J., Song Z., Urbanczyk-Wochniak E., Watson G. (2020). Environmental Fate and Dissipation of Applied DsRNA in Soil, Aquatic Systems, and Plants. Front. Plant Sci..

[105] European Food Safety Authority (EFSA) (2014). International Scientific Workshop ‘Risk Assessment Considerations for RNAi-based GM Plants’. EFSA Support. Publ..

[106] Health Canada Pest Management Regulatory Agency (PMRA) (2024). Information Note Regulation of dsRNA-Based Pesticides.

[107] Christiaens O., Whyard S., Vélez A.M., Smagghe G. (2020). Double-Stranded RNA Technology to Control Insect Pests: Current Status and Challenges. Front. Plant Sci..

[108] Levanova A.A., Poranen M.M. (2024). Utilization of Bacteriophage Phi6 for the Production of High-Quality Double-Stranded RNA Molecules. Viruses.

[109] Jiang X., Attiogbe K.B., Guo Y., Wu X. (2024). Production of Double-Stranded RNA Using the Prokaryotic Promoter-Mediated Bidirectional Transcription. Double-Stranded RNA: Methods and Protocols.

[110] Kim K., Kim S.H., Yoon K.A., Cho Y.S., Yoo M.-S., Lee S.H. (2018). Characterization of the Small Hive Beetle Transcriptome Focused on the Insecticide Target Site and RNA Interference Genes. J. Asia. Pac. Entomol..

